# Antibiotics that target mitochondria extend lifespan in *C. elegans*

**DOI:** 10.18632/aging.205229

**Published:** 2023-11-09

**Authors:** Gloria Bonuccelli, Darren R. Brooks, Sally Shepherd, Federica Sotgia, Michael P. Lisanti

**Affiliations:** 1 Translational Medicine, School of Science, Engineering and Environment (SEE), University of Salford, Greater Manchester M5 4BR, UK

**Keywords:** *C. elegans*, aging, lifespan, lipofuscin, antibiotics, mitochondria, metabolism, DPI

## Abstract

Aging is a continuous degenerative process caused by a progressive decline of cell and tissue functions in an organism. It is induced by the accumulation of damage that affects normal cellular processes, ultimately leading to cell death. It has been speculated for many years that mitochondria play a key role in the aging process. In the aim of characterizing the implications of mitochondria in aging, here we used *Caenorhabditis elegans* (*C. elegans*) as an organismal model treated a panel of mitochondrial inhibitors and assessed for survival. In our study, we assessed survival by evaluating worm lifespan, and we assessed aging markers by evaluating the pharyngeal muscle contraction, the accumulation of lipofuscin pigment and ATP levels. Our results show that treatment of worms with either doxycycline, azithromycin (inhibitors of the small and the large mitochondrial ribosomes, respectively), or a combination of both, significantly extended median lifespan of *C. elegans*, enhanced their pharyngeal pumping rate, reduced their lipofuscin content and their energy consumption (ATP levels), as compared to control untreated worms, suggesting an aging-abrogating effect for these drugs. Similarly, DPI, an inhibitor of mitochondrial complex I and II, was capable of prolonging the median lifespan of treated worms. On the other hand, subjecting worms to vitamin C, a pro-oxidant, failed to extend *C. elegans* lifespan and upregulated its energy consumption, revealing an increase in ATP level. Therefore, our longevity study reveals that mitochondrial inhibitors (i.e., mitochondria-targeting antibiotics) could abrogate aging and extend lifespan in *C. elegans*.

## INTRODUCTION

Aging is a continuous process which represents an inevitable decline in cell, tissue and organ functions, in almost all living organisms [1]. It is well established that numerous diseases are associated with aging, including diabetes, obesity, heart disease, neurodegeneration, and cancer. Slowing down aging has always been an ambitious aspiration, pursued by many biologists, geneticists, and the medical community.

Cellular senescence, considered as the basis of aging, represents a complex set of processes that are triggered during the aging of cells and tissues. Senescent cells are aging cells that have a permanent state of cell-cycle arrest, but do not undergo cell death. Indeed, these cells maintain a progressive and significant capacity to produce cytokines and inflammatory mediators [2, 3], thus influencing the surrounding environment and triggering chronic inflammation and fibrosis. In addition, senescence-mediated factors could stimulate the proliferation of adjacent cells, potentially promoting malignant transformation [4, 5]. Conversely, cellular senescence could have beneficial effects and is considered as a defense mechanism that is established in the aim of preventing malignant transformation, and suppressing tumorigenesis, causing irreversible growth arrest [6, 7].

Evaluating biological aging involves measuring many physiological features in humans or animals. For instance, the grip strength and heart rate are used as markers of aging and have been shown to be decreased in older organisms [8–10].

Among models used in life span studies is the *Caenorhabditis elegans* (*C. elegans*) nematode. Given its editable genome that shares high homology with that of mammals, its short life span allowing a better tracking of life phases, and ease of cultivation and storage, *C. elegans* has been efficiently used in scientific studies more specifically investigating the aging process [11, 12]. Contractions of the *C. elegans* pharynx, an organ composed of muscles and neurons, are correlated with worm life span, and have been evaluated in many studies by assessing the effect of many genetic or biochemical interventions [13, 14].

An important marker of senescence is the accumulation of lipofuscin, an “undegradable” pigment formed by misfolded proteins, lipids, and metal ions [15, 16]. In humans, an increased lipofuscin content is implicated in the development of the age-related macular degeneration (AMD) [17] and has been observed in the brains of Alzheimer’s disease patients (AD) [18]. In addition to humans, accumulation of this auto-fluorescent pigment is also described in various animal models, such as the mouse [19], the rat [19], zebrafish [20] and *C. elegans* [21, 22].

Mitochondria are organelles that have been shown to be directly implicated in the aging process. However, its exact role remains unclear. Mitochondrial malfunctioning observed during aging is associated with increased production of Reactive Oxygen Species (ROS) [23], the probable effectors of aging [24]. Indeed, in larger animals, such as humans [23], ROS are considered highly deleterious as they accelerate aging [25, 26]. On the other hand, in *C. elegans*, low levels of oxidative stress were sometimes shown to be associated with longevity [27, 28]. Intriguingly, studies have also demonstrated that inhibition of mitochondrial translation reduced respiration and extended lifespan in *C. elegans* [29].

In this study, we aim to test known compounds that target mitochondrial and metabolic activities, for their lifespan-increasing and health-improving effects using the animal model of *C. elegans*. Such compounds include the antibiotics doxycycline and azithromycin, diphenyleneiodonium chloride (DPI) (a metabolic inhibitor), and vitamin C. Our ultimate goal is to find existing FDA-approved drugs and dietary supplements that can, not only increase the lifespan, but also improve healthspan [30].

## RESULTS

### Effect of doxycycline on *C. elegans* aging

**Figure 1:**
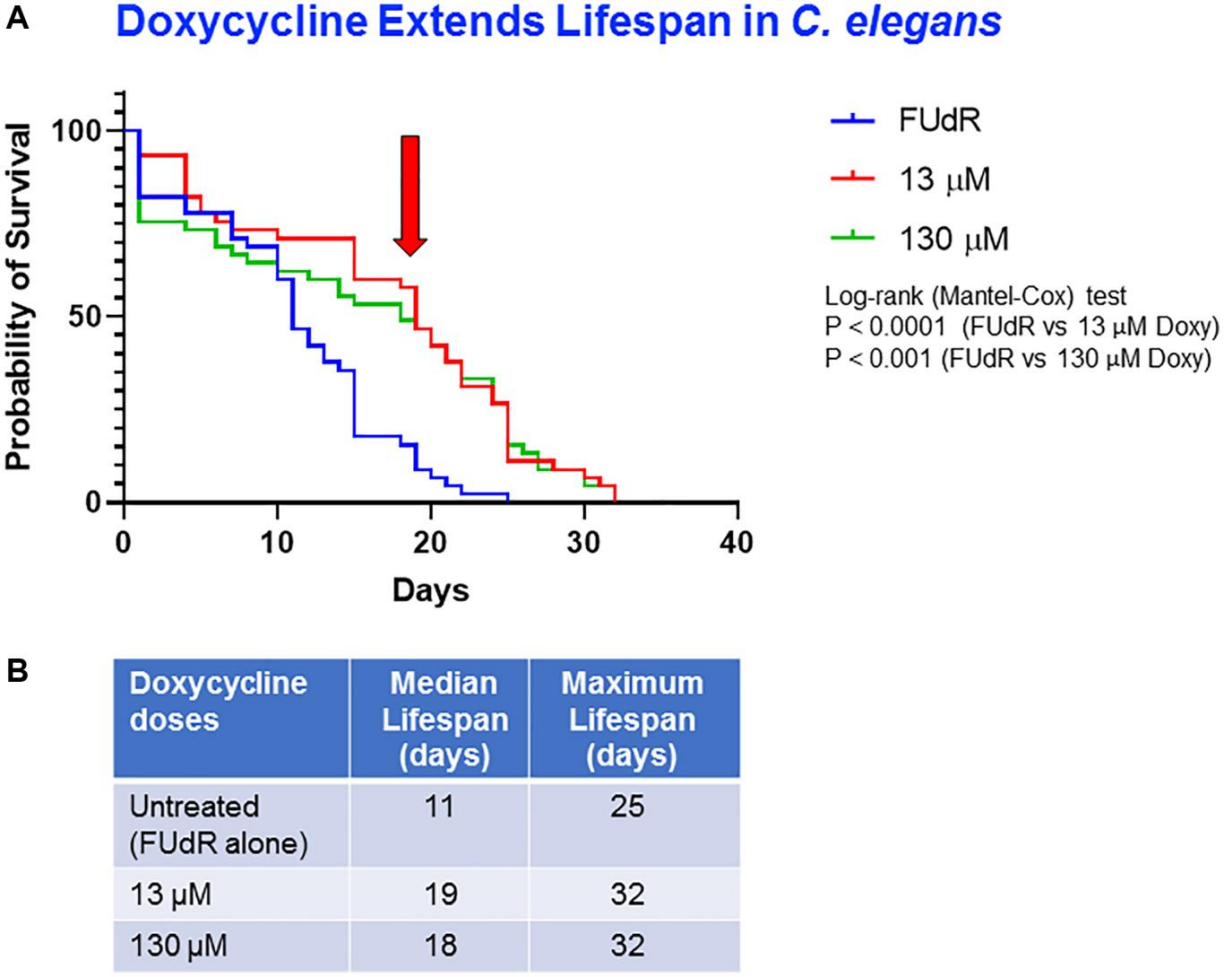
**Doxycycline extends lifespan of *C. elegans*.** (**A**) Survival curves of worms treated with different concentrations of doxycycline: control (FUdR alone) (blue), 13 μM doxycycline (red) and 130 μM doxycycline (green). Statistical analysis was performed using Log-rank (Mantel-Cox), ^****^p < 0.0001 (control vs. 13 μM), ^***^p < 0.001 (control vs. 130 μM). (**B**) Median survival and maximal lifespan are represented. Experiments were carried out in triplicate, with 15 worms for each replicate.

In order to establish evidence that the *C. elegans* model system works well in our hands, we started by reproducing previously published results. In the first set of experiments, we assessed the effects of the antibiotic doxycycline (an inhibitor of the small mitochondrial ribosome), previously demonstrated to extend the worm lifespan by phenocopying an *mrps-5* knockdown [29]. Here, we tested two concentrations: 13 μM (6 μg/ml) and 130 μM (60 μg/ml). Our survival curve data shows that treating young *C. elegans* adults with doxycycline at 13 μM and at 130 μM significantly increased their life span by 72.8% and 63.64%, respectively, as compared to the vehicle-alone controls (median lifespans: control = 11 days vs. treated with doxycycline 13 μM = 19 days or with 130 μM = 18 days) (Figure 1A, 1B). In addition, using the blue autofluorescence imaging (DAPI filter set) to evaluate the accumulation of the aging pigment lipofuscin in the worm, our results show that a 13 days treatment with doxycycline induced a dose-dependent decrease in *C. elegans* lipofuscin content of approximately by 50% in the worms treated with doxycycline 13 μM and by 90% in the worms treated with doxycycline 130 μM (Supplementary Figure 1). A further assessment of aging in *C. elegans* involved the evaluation of its pharyngeal muscle contraction. In this matter, treating young *C. elegans* adults with doxycycline for 14 days enhanced the worm’s rhythmic and sustained movement of the pharynx, suggestive of a longevity-inducing effect (Supplementary Video 1A–1C).

### Effect of azithromycin on *C. elegans* aging

**Figure 2:**
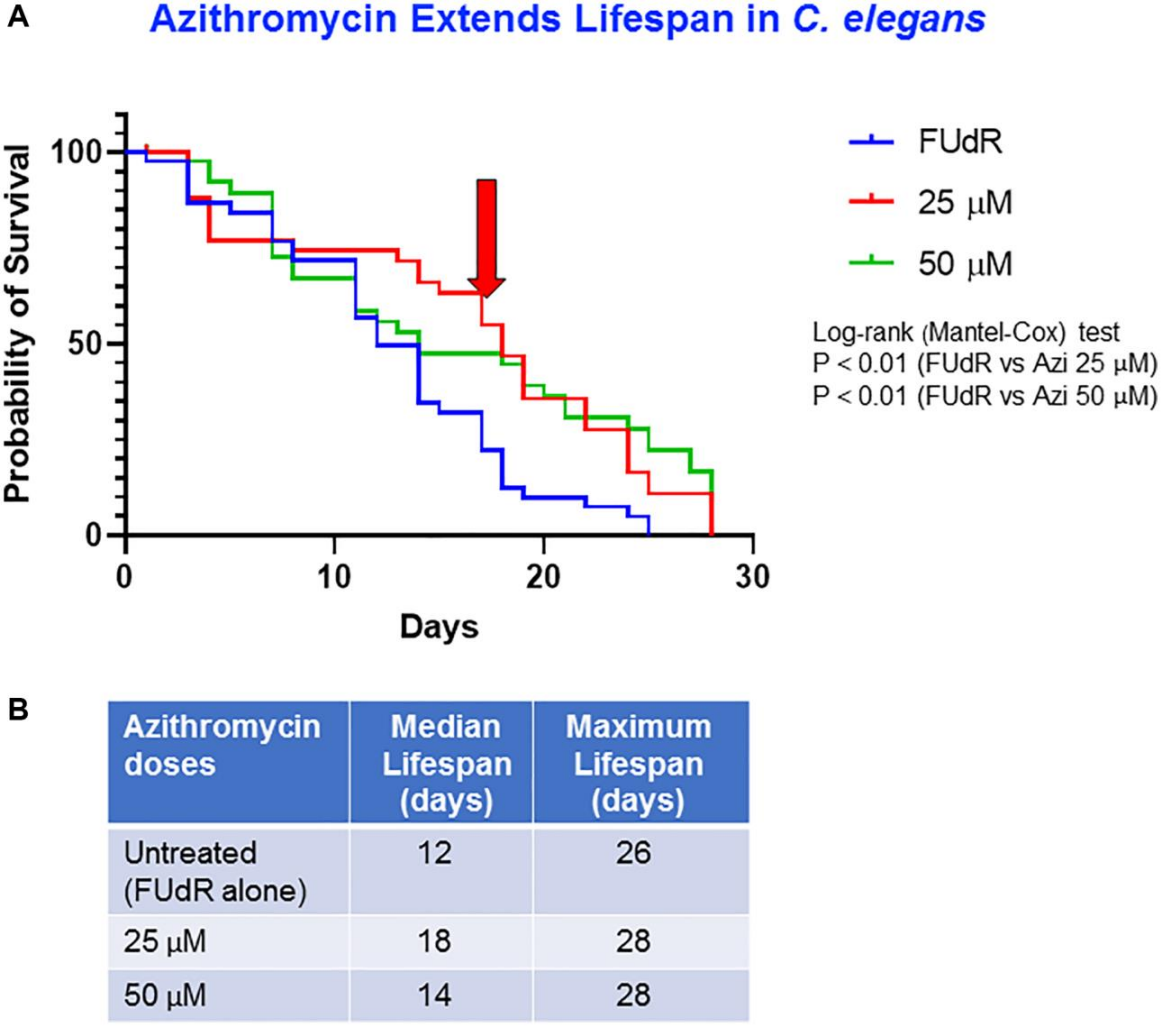
**Azithromycin extends lifespan.** (**A**) Survival curves at different concentrations of azithromycin: untreated control (FUdR-only) (blue), 25 μM (red) and 50 μM (green) AZI. Statistical analysis was performed using Log-rank (Mantel-Cox), ^**^p < 0.01 (control vs. 25 μM; control vs. 50 μM). (**B**) Median survival and maximal lifespan are represented. Experiments were carried out in triplicate, with 15 worms for each replicate.

In the next set of experiments, we extended our analysis to include another well-known antibiotic, namely azithromycin, an inhibitor of the large mitochondrial ribosome. Our survival curve results demonstrated that treating *C. elegans* with two different concentrations of azithromycin (25 μM and 50 μM) significantly increased their life span by 50% and 17%, respectively, as compared to the vehicle-alone-treated controls (median lifespans: control = 12 days vs. treated with azithromycin 25 μM = 18 days or with 50 μM = 14 days, Figure 2A, 2B). Qualitative assessment of lipofuscin showed that azithromycin slightly reduced lipofuscin accumulation at the dose of 25 μM and more markedly at the dose of 50 μM, compared to controls: approximately by 10% in the worms treated with azithromycin 25 μM and by 60% in the worms treated with azithromycin 50 μM (Supplementary Figure 2).

### Effect of diphenyleneiodonium on *C. elegans* aging

With the aim of inhibiting mitochondrial functionality using a different target, we next tested the possible effects of the well-known NADPH oxidases (NOX) enzymatic inhibitor, diphenyleneiodonium (DPI) [31], on *C. elegans* life span. NADPH oxidases are responsible for the production of cellular reactive oxygen species (ROS) such as superoxide and hydrogen peroxide [32], known effectors of aging. In this matter, several evidences have demonstrated that controlling the activity of NADPH oxidases in cellular homeostasis is fundamental for a healthy aging [33].

**Figure 3:**
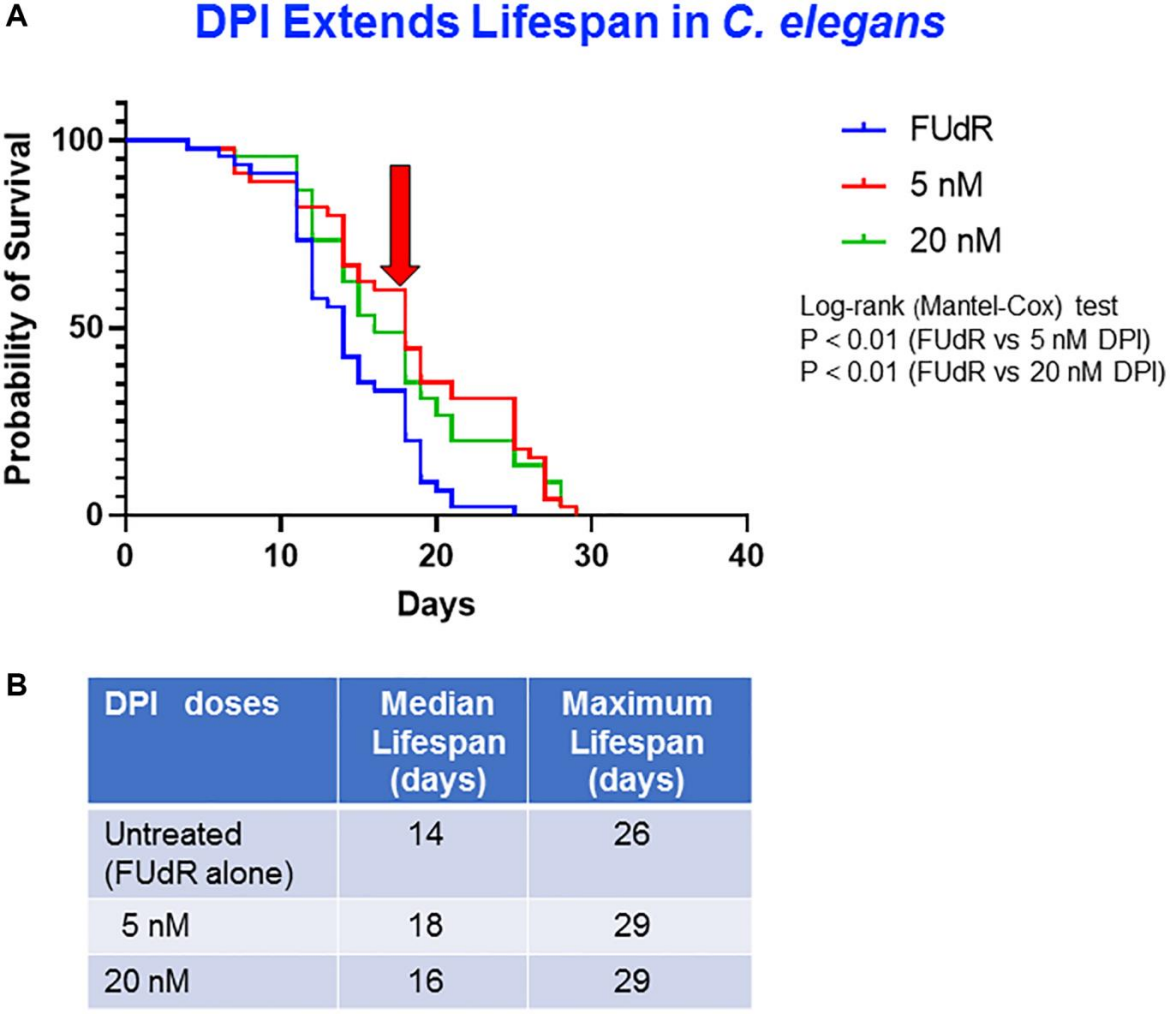
**DPI extends lifespan of *C. elegans*.** (**A**) Survival curves at different concentrations of DPI: untreated control (FUdR alone) (blue), 5 nM (red) and 20 nM (green). Statistical analysis was performed using Log-rank (Mantel-Cox), ^**^p < 0.01 (control vs. 5 nM; control vs. 20 nM). (**B**) Median survival and maximal lifespan are represented. Experiments were carried out in triplicate with 15 worms for each replicate.

Based on our previous validation experiments of DPI as a potent mitochondrial inhibitor in breast cancer cells [31], we chose to evaluate herein two DPI concentrations, 5 nM and 20 nM. Interestingly, a significant increase in the median lifespan was observed in the treated worms as compared to the vehicle controls (median lifespans: control = 14 days vs. treated with DPI 5 nM = 18 days or with 20 nM = 16 days) (Figure 3A, 3B). Next, we acquired images with the DAPI filter to evaluate the lipofuscin content. We observed only a slight reduction of lipofuscin of approximatively 10% in the 20 nM treated worms compared to controls (Supplementary Figure 3).

### Combination therapy and its effect on *C. elegans* aging

We have recently shown that combinations of low concentrations of doxycycline and azithromycin together with vitamin C effectively target and inhibit the propagation of breast cancer stem cells (BCSCs) which were: 1 μM doxycycline, 1 μM azithromycin and 250 μM vitamin C [34]. Here, we aimed to assess if the same combination was effective against aging in *C. elegans*, by prolonging its lifespan. We utilized the above agents at concentrations reported to efficiently inhibit MCF7 ER(+) breast cancer cells propagation (by >90%) [34].

**Figure 4:**
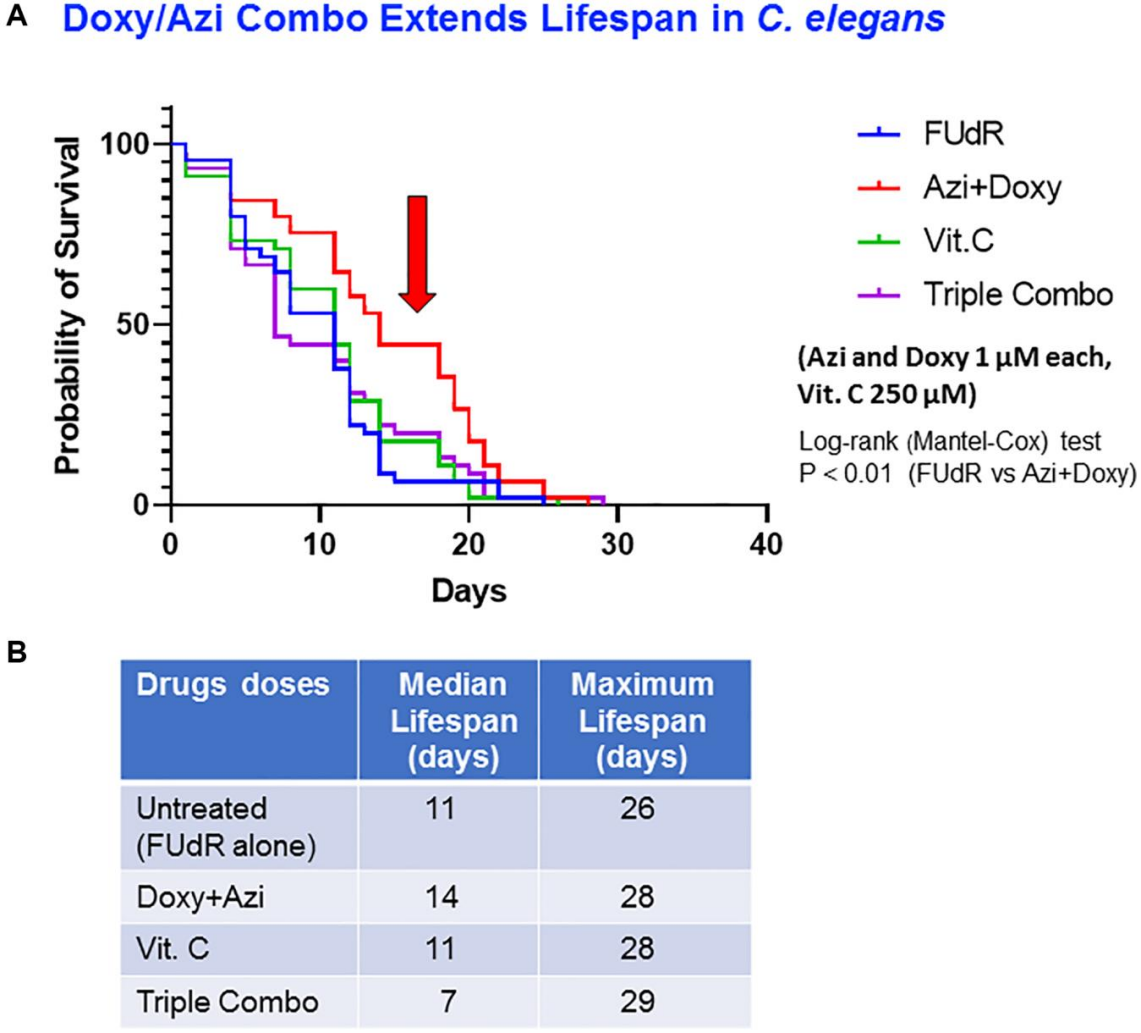
**A double combination, of doxycycline plus azithromycin, extends life span of *C. elegans*.** (**A**) Survival curve at different concentrations of antibiotics: untreated control (FUdR-only) (blue), Azi plus Doxy (1 μM each) (red), vitamin C alone (250 μM) (green) and triple combination (Azi plus Doxy plus vitamin C) (purple). Statistical analysis was performed using Log-rank (Mantel-Cox), ^**^p < 0.01 (control vs. Doxy plus Azi). (**B**) Median survival and maximal lifespan are represented. Experiments were performed in triplicate, with 15 worms for each replicate.

Worms were divided into four groups: 1) control worms; 2) worms treated with the combination of 1 μM doxycycline and 1 μM azithromycin; 3) worms treated with 250 μM vitamin C alone; and 4) worms treated with the triple combination of 1 μM doxycycline, 1 μM azithromycin and 250 μM vitamin C (Figure 4A). Results show that a significant survival (27% lifespan increase) was only observed in the group of worms treated with the combination of antibiotics (doxycycline and azithromycin), group 2, compared to controls (median lifespans: group 1 = 11 days; group 2 = 14 days, group 3 = 11 days and group 4 = 7 days) (Figure 4B).

In addition, the body movement and pharyngeal pumping of worms in the 4 groups was recorded after 14 days of treatment. Our data show that worms treated with vitamin C alone exhibited reduced velocity of body movement and pharyngeal pumping as compared to all other treatment groups and to vehicle controls.

Importantly, worms treated with the double and triple combinations demonstrated a relatively high pharyngeal pumping rate (Supplementary Video 2A–2D). Following a 19 days treatment with vitamin C alone, worms appeared similar to control worms or dead (Supplementary Video 3A, 3B). Conversely, worms treated for 19 days with the double combination of doxycycline plus azithromycin were capable of maintaining body movement and a slight pharyngeal contraction (Supplementary Video 3C, 3D). However, worms subjected to a 19 days treatment with the triple combination of doxycycline, azithromycin and vitamin C presented some body movement, but no signs of pharyngeal pump contractions. Furthermore, we visualized at different time points the fluorescent lipofuscin content in the various treatment groups defined above. Our data show a progressive increase in lipofuscin content in the vehicle-treated group (control) and in worms treated with vitamin C at day 8 (Supplementary Figure 4A) and day 13 (Supplementary Figure 4B) post-treatment of approximately 18% for both, whereas lipofuscin did not accumulate in worms treated with the double or triple combination of the above agents, showing an accumulation percentage reduced by approximately 40% (day 8) and 30% (day 13) compared to the untreated ones (Supplementary Figure 4A, 4B).

### Evaluating ATP levels in *C. elegans*

**Figure 5:**
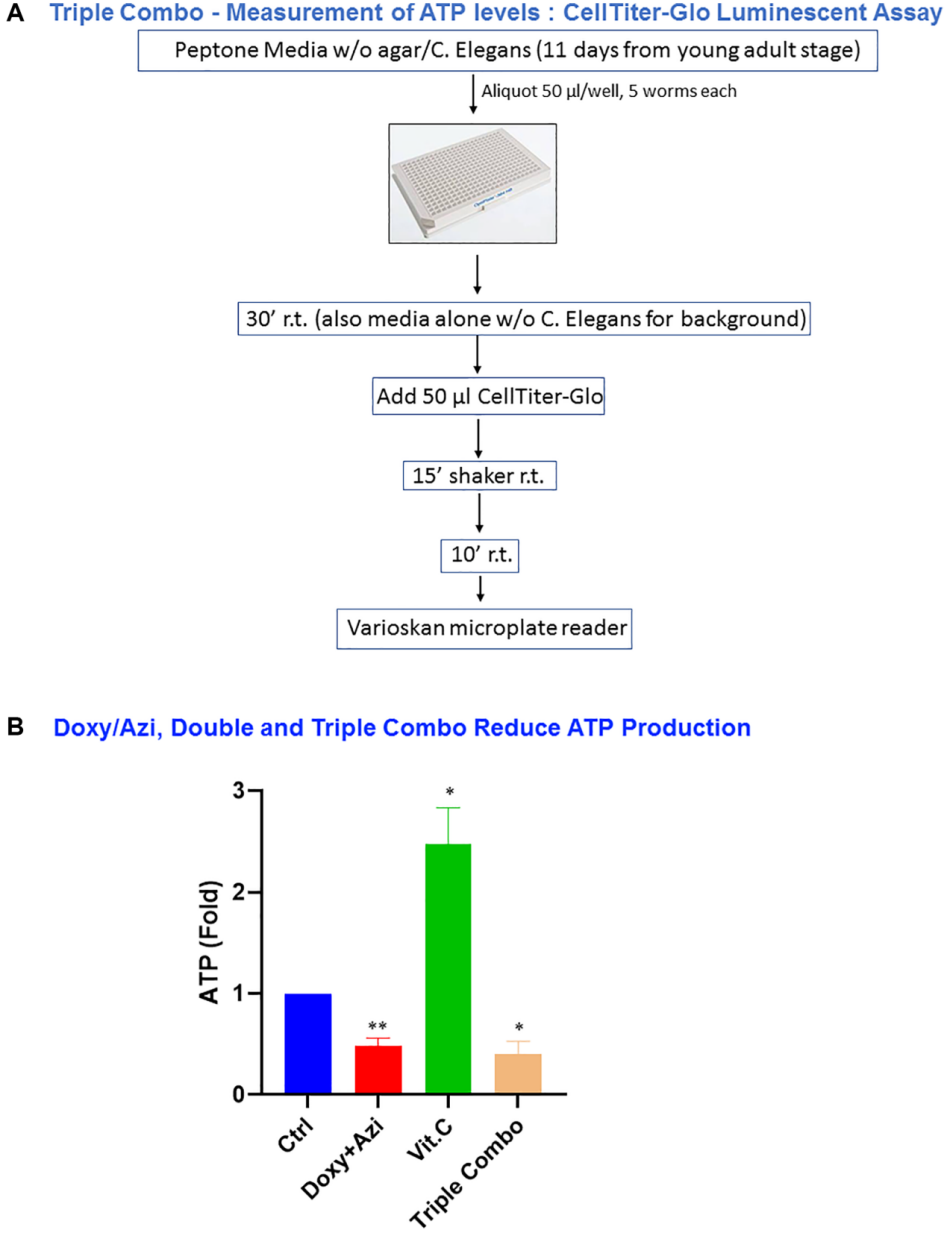
**The double combination, doxycycline plus azithromycin, and a triple combination, doxycycline plus azithromycin plus vitamin C, were effective in decreasing the ATP levels in *C. elegans*.** (**A**) Schematic representation of the protocol for CellTiter-Glo luminescent assay performed in experiments using the double and triple combination. (**B**) Measurement of ATP levels. The final concentrations of the compounds used in this experiment were 1 μM for doxycycline and azithromycin, and 250 μM for vitamin C. Data are shown as the mean ± SEM; Statistical analysis was performed using one-sample *t*-test. ^*^p < 0.05, ^**^p < 0.01. Experiments were repeated 4 times, using 5 worms for each condition.

To characterize the mechanism by which mitochondrial inhibitors extend lifespan, we measured the levels of ATP content upon treating worms with the various combinations of antibiotics and vitamin C. To this aim, we modified the protocol of the CellTiter-Glo assay generally used as ATP-based cell viability assay, as schematized in Figure 5A (see Materials and Methods for detailed description). The CellTiter-Glo assay is based on the quantification of ATP content, reflecting metabolically active cells. Intriguingly, the double combination (doxycycline plus azithromycin), as well as the triple combination (doxycycline plus azithromycin plus vitamin C) were significantly effective in decreasing the ATP levels by 50%, as compared to controls. Conversely, vitamin C alone, which is a known pro-oxidant, caused a significant increase in the production of ATP of more than 2.5 fold (Figure 5B).

## DISCUSSION

The goal of delaying or halting aging has been strong desire of humans for many centuries. Scientists have long been investigating many aspects of aging and are pursuing a diligent search for anti-aging therapies. Aging is a risk factor of many diseases, ranging from heart disease and diabetes to dementia and cancers [35]. Indeed, targeting aging aims not only at increasing life expectancy but also at enhancing healthspan, the heathy years of life. Enhancing homeostasis-related physiological processes should reverse aging and its associated biological mechanisms. In this regard, many natural or synthetic molecules have been evaluated for their potential anti-aging effects and have shown various levels of efficiency [36]. In this study, we aimed to examine the effects of different compounds on the lifespan of *C. elegans* worms.

Given the crucial role of the mitochondria in cellular processes and the fact that mitochondrial dysfunction is associated with cell senescence and aging [37, 38], we chose to investigate whether compounds that inhibit mitochondrial oxygen consumption and ATP production, effect lifespan and healthspan using the *C. elegans* model. We demonstrated that the antibiotic doxycycline, which is an inhibitor of the small subunit of the mitochondrial ribosome, increases the worm lifespan. This is in line with evidence published previously, which showed that doxycycline treatment induces a dose-dependent increase in mean lifespan [29]. Consistently, we have confirmed the ability of doxycycline at 6 μg/ml and 60 μg/ml to extend the lifespan as compared to control worms, but we have not observed significant differences between the two doses tested as seen by Houtkooper et al. [29]. This discrepancy could be due to procedural variabilities between different laboratories, such as small temperature fluctuations in incubators, and differences in food composition [39]. It is worth noting here that the lowest concentration of doxycycline used in this set of experiments, 6 μg/ml (13 μM), is within the range of the maximum plasma levels of doxycycline achievable in patients following the administration of a normal daily dose of 200-mg, corresponding to 3–5 μg/ml [40]. Importantly, our results also demonstrated that doxycycline induced a dose dependent decrease in the accumulation of the aging-pigment lipofuscin, together with a more pronounced pharyngeal contraction activity, indicating that this mitochondria-targeted drug promotes a more vital and healthier state in *C. elegans*. Our results are concomitant with other mitochondrial-mediated healthy span-inducing effects. Indeed, our group has previously demonstrated that doxycycline blocks 3D mammosphere formation of various malignant cell lines, with an IC-50 between 2-to-10 μM [41, 42]. Furthermore, doxycycline was shown to reduce markers of CSCs in samples of breast cancer tumors [43]. Taken together, these data highly suggest the efficiency of doxycycline in increasing life span and enhancing health quality.

Another antibiotic, azithromycin that targets the mitochondrial ribosome machinery and inhibits mitochondrial translation as an “off-target”, is evaluated herein as to its lifespan-mediating actions. Azithromycin is an FDA-approved antibiotic belonging to the class of erythromycins [41]. We tested azithromycin at two concentrations on worms, 25 μM and 50 μM that translated in clinical practice as 18.72 μg/ml and 37.44 μg/ml, respectively (higher than the achievable concentration in patient’s serum upon oral azithromycin 500 mg: 0.39 μg/ml [44]). Azithromycin, especially at the lower concentration used, was capable of significantly extending the worms lifespan. Our data on both antibiotics are consistent with previously reported data by our group, demonstrating their capacity to interfere with the mitochondrial biogenesis in CSCs of various origin [41]. Indeed, Azithromycin was also shown by our group to selectively eliminate senescent cells, thus functioning as a clinically approved drug, with senolytic properties [45].

Next, we wanted to study the inhibition of the mitochondrial oxidative process using DPI, a compound that potently abrogates the flavin-containing (FMN and FAD-dependent) subunits of Complex I and II of the respiratory chain. We have previously demonstrated that DPI blocks the propagation of breast CSCs, while inhibiting their oxidative mitochondrial metabolism (OXPHOS) and decreasing mitochondrial driven ATP production by >90% [31]. More recent studies revealed an anti-tumoral effect of DPI inducing senescence or apoptosis of colorectal and breast cancer cells depending on their p53 expression [46].

As to *C. elegans* studies in this matter, since DPI inhibits oxidative damage in the worms, it was also shown to reduce accumulation of the age pigment, lipofuscin in *C. elegans*, indicative of increased longevity [47]. However in the latter study, *C. elegans* fed on *E. feacalis*, a pathogenic bacterium that induces the worm killing, exhibited a reduced lifespan upon its treatment with DPI probably due to the fact that DPI interfered with the worm oxidative anti-bacterial defense mechanisms increasing its sensitivity to the pathogenic bacteria [47]. In contrast with these results, but consistent with our data, recent results showed that DPI can function as a senomorphic drug, reducing the number of beta-gal positive senescent cells *in vitro*. More importantly, DPI treatment of old mice decreased the number of senescent cells, reduced the expression of inflammatory mediators and ameliorated signs of aging, such as liver fibrosis and immune cell infiltration, and improved physical performance [48]. Overall, these data support the idea that DPI can extend lifespan and reverse aging in mammalians as well as *C. elegans*. All of these data are coherent with the well-established longevity-promoting effects of anti-oxidants, decreasing the production of oxygen free radicals and other reactive oxygen species (ROS) and abrogating their deleterious effect on cell survival [49, 50]. Interestingly, the protective effect we observed for DPI against aging was obtained at very low concentrations thus with little if any toxicity produced, an added value to its lifespan-increasing function.

Subsequently, we evaluated combination treatments using more than one antibiotic but at lower concentrations, in the aim of increasing efficacy. Indeed, our double antibiotic combination (doxycycline-azithromycin, DOXY-AZI) included concentrations of 1 μM (0.5 μg/ml) of doxycycline and 1 μM (0.75 μg/ml) of azithromycin. The maximum concentration of azithromycin in the plasma of a patient receiving a normal daily dose of 500 mg or a multiple dose regimen of azithromycin is 0.4 μg/ml. As a consequence, the dose of azithromycin we used for these longevity experiments on worms is within the range of an achievable dose in an adult patient. Our results demonstrated that the combination of antibiotics at low concentrations, was also capable of significantly extending the median lifespan of *C. elegans*, and maintaining their pharyngeal muscle activity as compared to controls. In addition, such combination treatment downregulated the aging process, as shown by the reduced level of lipofuscin accumulation in treated worms and reduced mitochondrial function assessed by a decreased ATP consumption. These data are coherent with our previous report of the mitochondrial dysfunction effect of the DOXY-AZI combination, whereby treating breast CSCs with the combination abrogated their mitochondrial oxygen consumption and reduced their ATP levels [34].

Interestingly, adding vitamin C to the antibiotic combination conserved its anti-aging effect (maintained pharyngeal activity and decreased lipofuscin content), but it reversed the lifespan-extending effects of the antibiotic combination (DOXY-AZI). We have previously evaluated the effects of this antibiotic + Vitamin C combination on CSCs and shown that it inhibited the propagation of breast CSCs by more than 90%, abrogated their mitochondrial activity and reduced their ATP levels. Vitamin C, the known pro-oxidant and free radicals producer, while inducing mitochondrial biogenesis in cells, will expose mitochondria to the inhibitory action of the mitochondrial-targeting antibiotics, doxycycline and azithromycin, rendering the effect of a combination treatment with the three agents more efficient in abrogating the stemness and propagation of cancer cells. Thus, data from our present study showing that a triple combination treatment (DOXY-AZI-Vit.C) while still exhibiting some anti-aging effect, could not really extend life span of *C. elegans*, suggest that Vitamin C does not add to the beneficial effect of the double combination DOXY-AZI, at least in the extension of lifespan.

In summary, we have identified some mitochondrial inhibitors for the extension of lifespan in the animal model of *C. elegans*. This supports the theory that mitochondria are heavily involved in the aging process, although this remains a highly debated topic. Intriguingly, the compounds used in this study are for the most part repurposed agents for which preclinical and clinical studies have already been performed to establish their low toxicity.

## MATERIALS AND METHODS

### Materials

Doxycycline, DPI (Diphenyleneiodonium chloride) and L-ascorbic acid (Vitamin C) were purchased from Sigma-Aldrich. Azithromycin was purchased from Tocris Bioscience™ and CellTiter-Glo luminescent assay was from Promega.

### Strain maintenance

N2 (Bristol) strain worms were grown at 20°C on standard nematode growth medium (NGM) plates using standard techniques [51] and seeded with *Escherichia coli* OP50. Plates were maintained in an incubator at 20°C. As an invertebrate, *C. elegans* research is considered of no ethical concern and hence ethical clearance is granted.

### Lifespan analysis

To acquire an age-synchronized population of animals, 10 to 15 reproductively active adults were transferred to a fresh NGM plate and incubated at 20°C for 8 hours to allow time to lay eggs. Then, adults were removed from those plates and plates were monitored until eggs hatched and animals developed to the young adult stage (about 2 days). These young adult worms were then transferred to NGM plates containing 25 μM of 5-fluorodeoxyuridine (FUdR), an inhibitor of DNA synthesis. Importantly, the laid eggs in the presence of FUdR do not hatch, which maintains the synchrony of the culture. In more detail, 15 worms were individually picked and seeded onto three freshly prepared solid media plates, all supplemented with FUdR to a final concentration of 25 μM. In addition, the experimental groups (*N* = 15) were supplemented with the drugs of interest at the desired final concentration, in the presence of FUdR. Each experiment comprised the control group (only FUdR and the vehicle alone in which the compound was dissolved e.g., DMSO) and the treated groups (FUdR plus test compound). Worms under test conditions were fed with heat-killed bacteria OP50. Each experiment was repeated 3 times with three independent biological repeats. Worms were scored by gently tapping the plates, and the date and the number of worms that were alive and dead was recorded. Worms that crawled off the plates were disregarded from the analysis. Statistical analysis for survival was conducted using the standard χ^2^-based log rank-test.

### ATP assay

CellTiter-Glo luminescent assay was performed to measure metabolic activity (ATP content) in worms treated with a given compound. Assays were performed in 96-well plates, with five worms in each well, in 50 μl of liquid NGM media. Assays were carried out on 11-day-old adults. *C. elegans* were incubated in media for 30 minutes at room temperature (wells with media alone without *C. elegans* were used for calculating the background). Then, 50 μl of CellTiter-Glo was added to each well; plates were incubated for 15 minutes on a shaker at room temperature then allowed to rest for 10 minutes at room temperature. Luminescence intensities (correlating with ATP content) were measured using the Varioskan™ microplate reader, normalized to vehicle-alone treated controls and ATP values were displayed as percentages.

### Autofluorescence measurement for lipofuscin content

Photos were taken under a DAPI filter set using the EVOS Cell Imaging Systems (at 8 and/or 13 days from young adult stage). Pictures were taken at the same exposure and the fluorescent levels were identically adjusted, with the exact same contrast/brightness values for all paired photos, using ImageJ software.

### Locomotion videos

On solid media plates, worms were recorded using the EVOS Cell Imaging Systems to evaluate their body movements and the pumping rate of their pharynx (at 13 and/or 19 days from young adult stage).

### Statistical analysis

The log-rank test, equivalent to the Mantel-Cox method, was calculated by GraphPad Prism 8.0 for comparing the survival curve of control worms with the treated ones. *P*-values of less than 0.05 were considered significant. The median survival, determined using GraphPad Prism 8.0, was the length of time from the start of treatment that half of the worms in a group were still alive, compared to the untreated worms. The one-sample *t*-test was used in the ATP assay. Statistical analysis and plotting of the data were performed using GraphPad Prism 8.0 and Microsoft Excel.

## Supplementary Materials

Supplementary Figures

Supplementary Video 1A

Supplementary Video 1B

Supplementary Video 1C

Supplementary Video 2A

Supplementary Video 2B

Supplementary Video 2C

Supplementary Video 2D

Supplementary Video 3A

Supplementary Video 3B

Supplementary Video 3C

Supplementary Video 3D
