# Supplementary material for: Antibiotics that target mitochondria extend lifespan in C. elegans
Source: Aging (Albany NY). 2023 Nov 9;15(21):11764–81. doi: 10.18632/aging.205229 (PMC10683609; doi:10.18632/aging.205229)
Supplement: Supplementary Figures [file aging-15-205229-s001.pdf]

SUPPLEMENTARY FIGURES

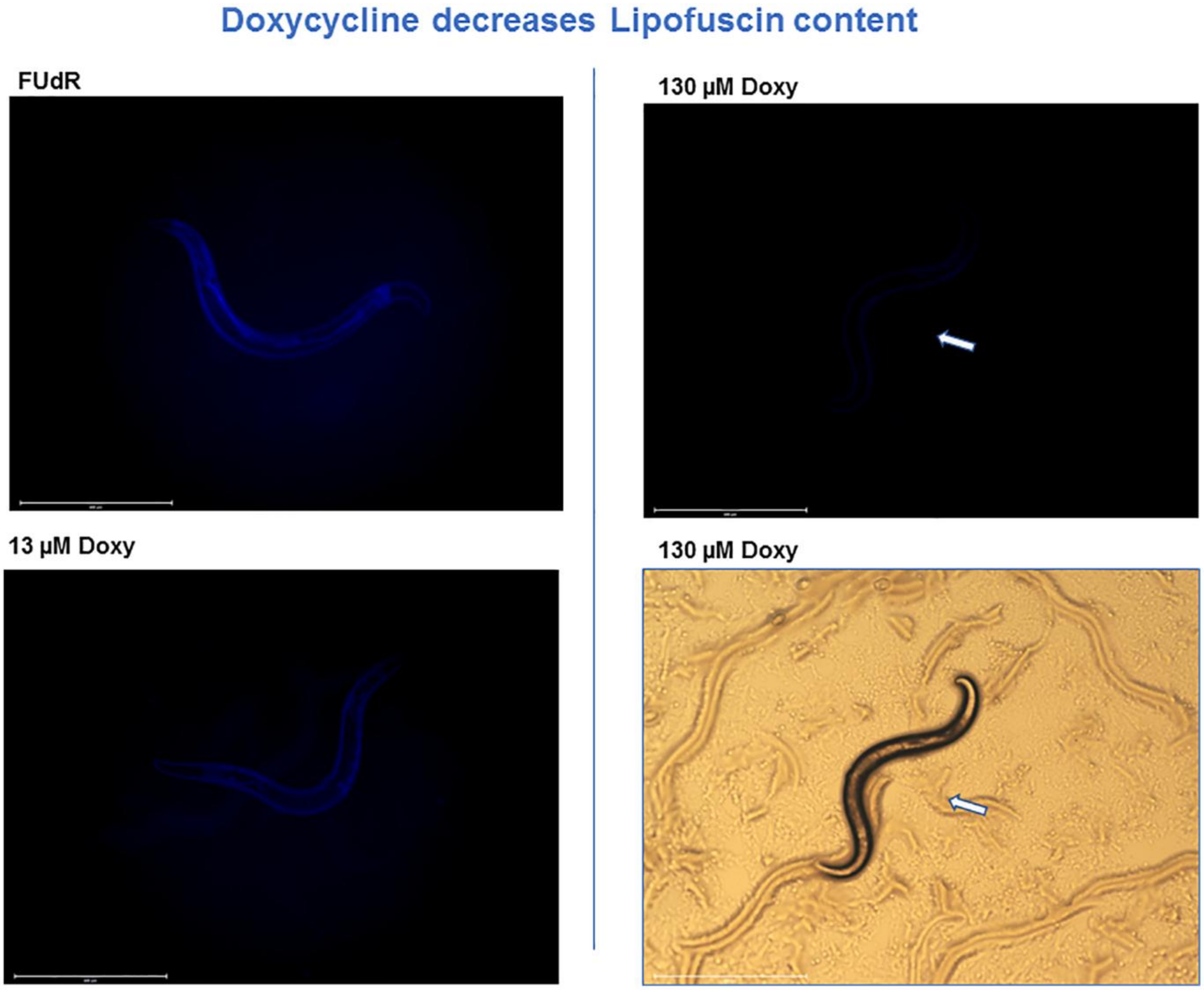

Supplementary Figure 1. Doxycycline decreases lipofuscin content in adults (13 days from young adult stage). A representative image of lipofuscin content evaluated by autofluorescence. DAPI filter set was used. Left half of the figure: top panel, FUdR control; lower panel, treatment with doxycycline at the concentration of 13 µM. Right half of the figure: top panel, treatment with doxycycline at the concentration of 130 µM; lower photograph as the one above but in brightfield. The arrow indicates the worm. Magnification 10X. Scale bar: 500 µm.

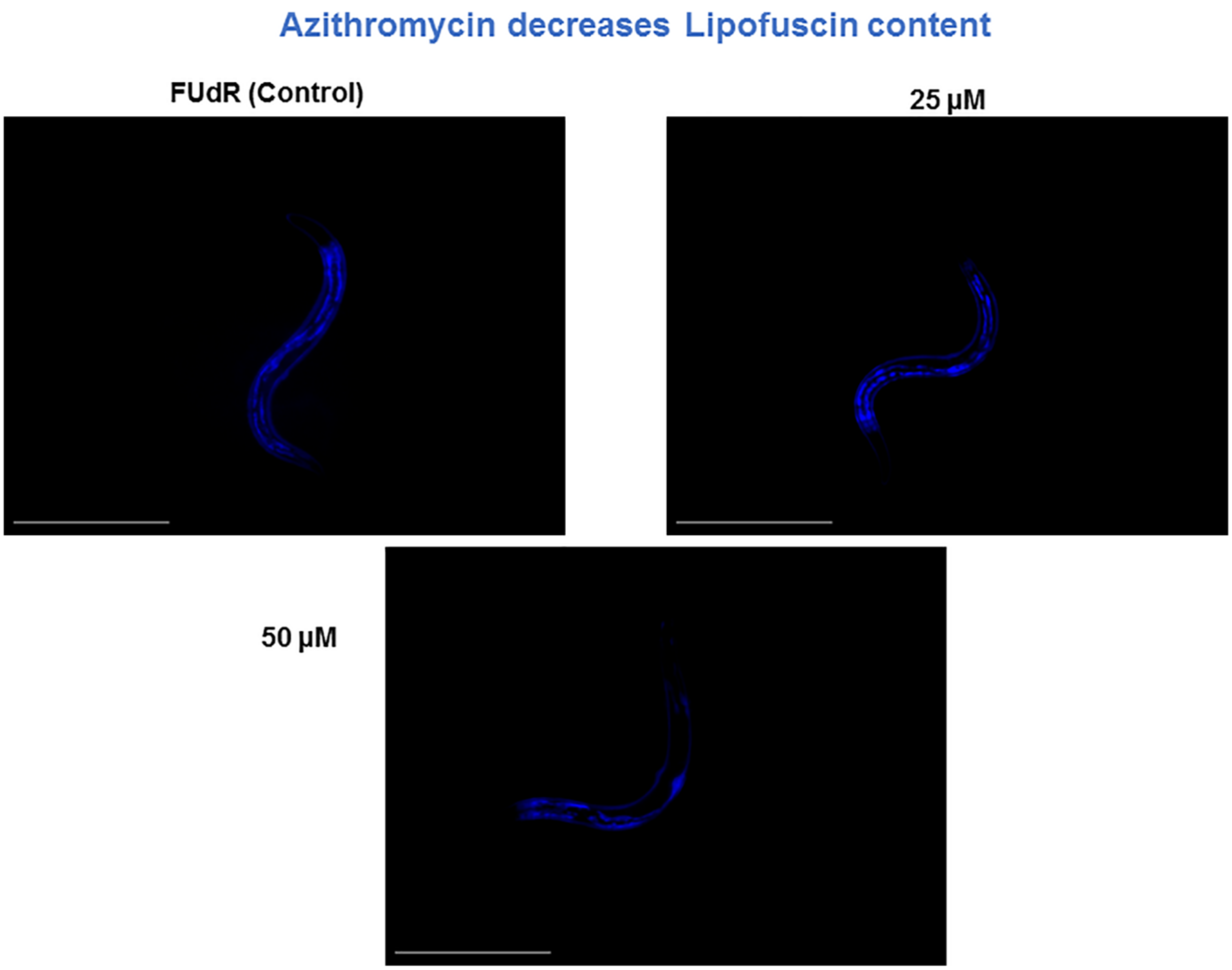

Supplementary Figure 2. Azithromycin decreases lipofuscin content in adults (13 days from young adult stage). Representative image of lipofuscin content evaluated by autofluorescence of the above pigment. DAPI filter set was used. Clockwise: FUdR control; *C. elegans* treated with azithromycin at the concentration of 25 µM; *C. elegans* treated with azithromycin at the concentration of 50 µM. Magnification 10X. Scale bar: 500 µm.

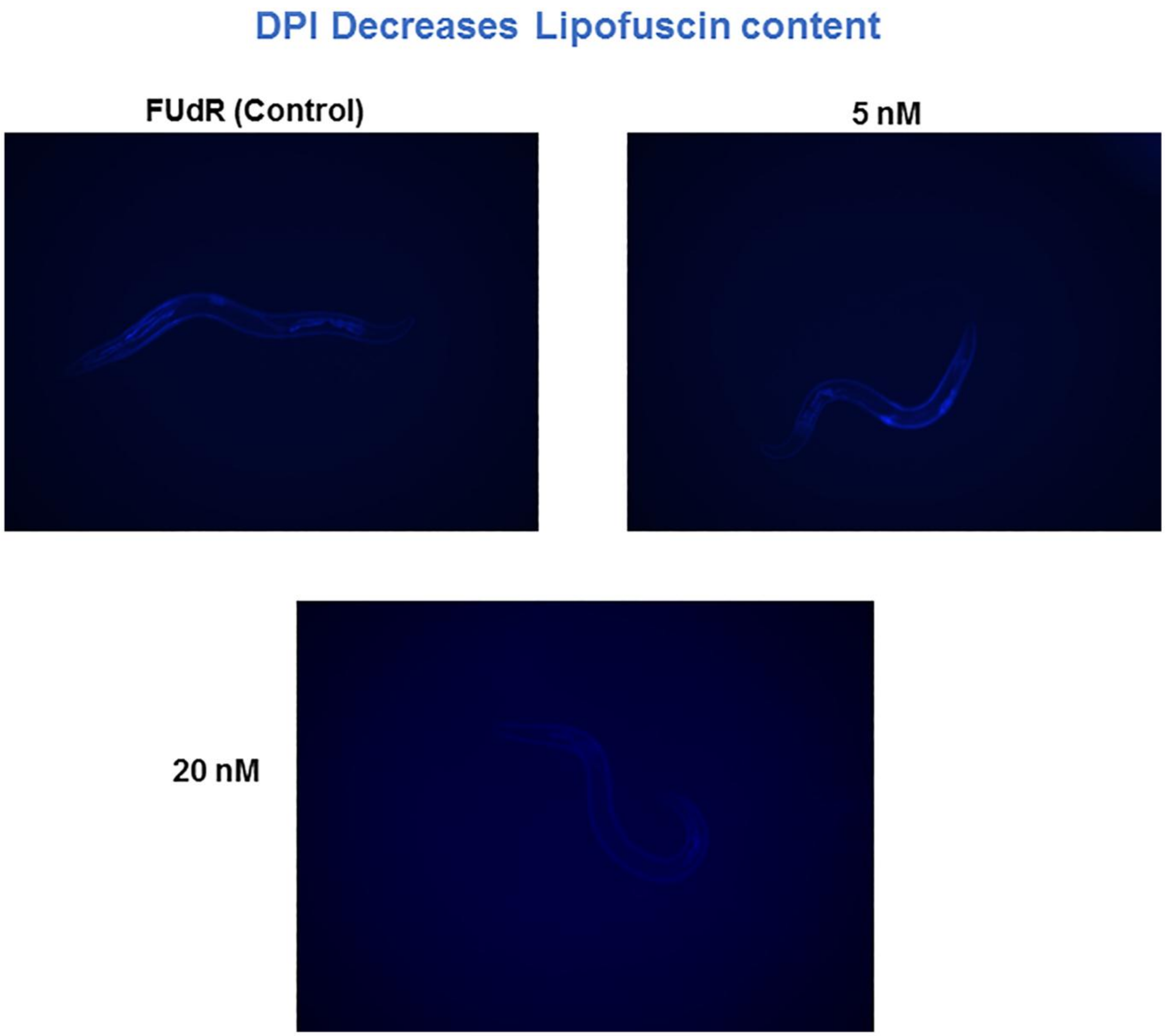

Supplementary Figure 3. DPI decreases lipofuscin content in adults (13 days from young adult stage). Representative image of lipofuscin content evaluated by autofluorescence of the above pigment. DAPI filter set was used. Clockwise: FUdR control; C. elegans treated with DPI at the concentration of 5 nM; *C. elegans* treated with DPI at the concentration of 20 nM. Magnification 10X. Scale bar: 500 µm.

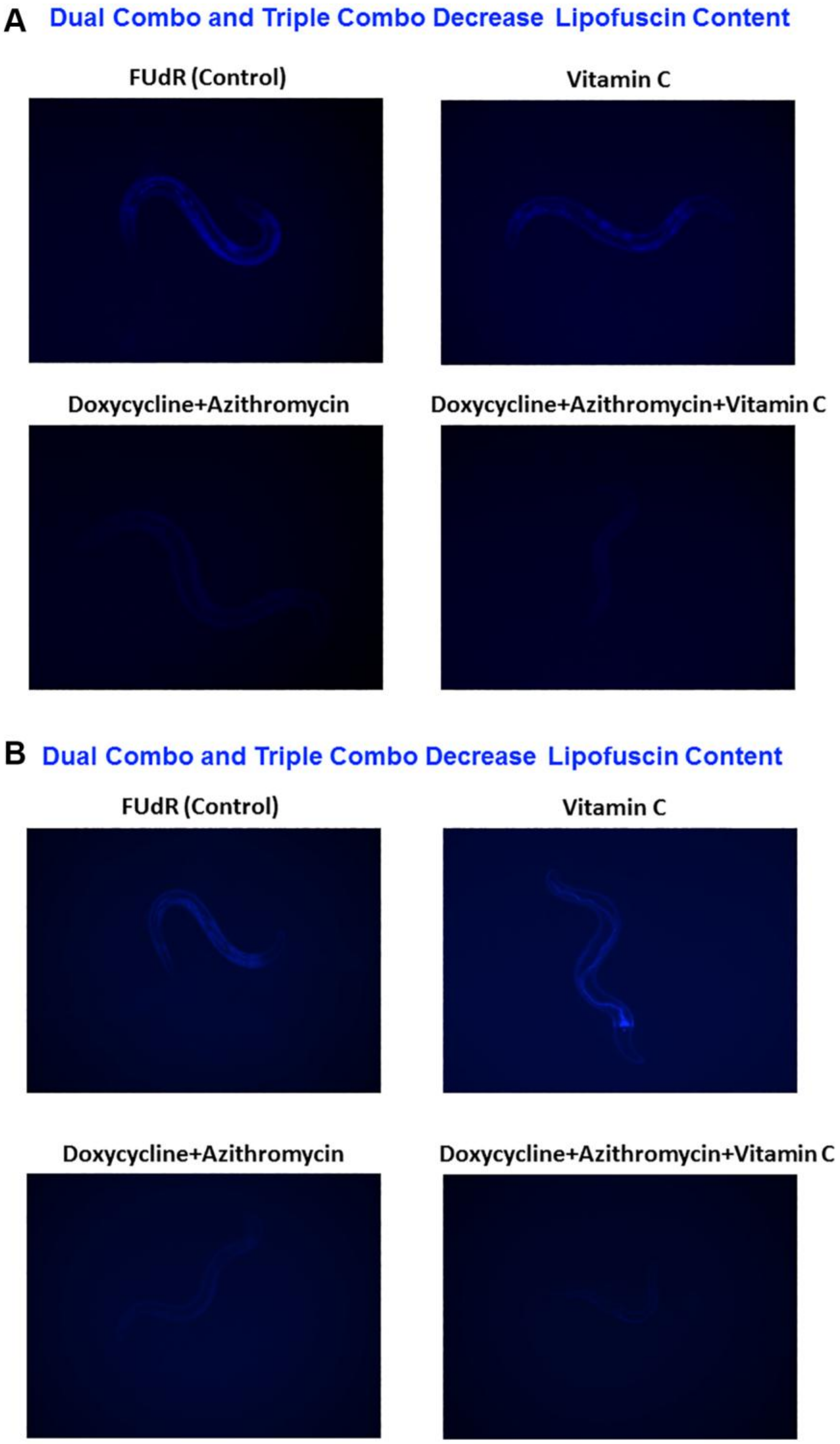

Supplementary Figure 4. Doxycycline/Azithromycin combination decreases lipofuscin content in adults. Representative images of lipofuscin content evaluated by autofluorescence of the above pigment. DAPI filter was used. (**A**) Evaluation at 8 days from young adult stage. (**B**) evaluation at 13 days from young adult stage. Clockwise: untreated control; *C. elegans* treated with vitamin C alone; *C. elegans* treated with doxycycline plus azithromycin plus vitamin C; *C. elegans* treated with doxycycline plus azithromycin. Magnification 10X. Scale bar: 500 µm.
